# Examining the Association Between Frequency of Mobile Clinic Visits and Diabetes and Hypertension Control

**DOI:** 10.3390/ijerph23030303

**Published:** 2026-02-28

**Authors:** Angela Coaston, Caroline Stephens, Soo-Jeong Lee, Sandra J. Weiss, Julene Johnson, Thomas Hoffmann

**Affiliations:** 1 School of Nursing, College of Health Science, Pepperdine University, Malibu, CA 90265, USA; 2 Community Health Systems, School of Nursing, University of California, San Francisco, CA 94158, USA; caroline.stephens@ucsf.edu (C.S.); soo-jeong.lee@ucsf.edu (S.-J.L.); 3 Gerontology, College of Nursing, University of Utah, Utah, UT 84112, USA; 4 Cognitive Neuroscience & Medicine, School of Medicine, University of California, San Francisco, CA 94158, USA; julene.johnson@ucsf.edu; 5 Epidemiology & Biostatistics, School of Medicine, University of California, San Francisco, CA 94143, USA; thomas.hoffmann@ucsf.edu

**Keywords:** mobile medical clinic, diabetes, hypertension, utilization, predisposing, enabling, need factors

## Abstract

**Highlights:**

**Public health relevance—How does this work relate to a public health issue?**

- Chronic diseases such as hypertension and diabetes disproportionately affect marginalized and underserved adults with limited access to traditional healthcare, making disease control a significant public health challenge.
- Mobile medical clinics are a community-based healthcare delivery model designed to reduce access barriers and address unmet health needs in underserved communities.

**Public health significance—Why is this work of significance to public health?**

- This study provides evidence that increased frequency of mobile clinic visits is associated with improved hypertension control over time, demonstrating the population-level value of mobile clinics in chronic disease management.
- By identifying differential effects of mobile clinic visit frequency on hypertension versus diabetes control, the findings highlight gaps in current care models and the need for diagnosis-specific public health interventions.

**Public health implications—What are the key implications or messages for practitioners, policy makers and/or researchers in public health?**

- Public health practitioners and healthcare systems should incorporate mobile medical clinics into chronic disease prevention and management strategies while integrating additional supports, such as nutrition education and physical activity interventions, to improve diabetes outcomes.
- Policy makers and researchers should advance value-based reimbursement approaches and cross-sector partnerships that include mobile clinics to improve access, reduce health disparities, and support sustainable community-based healthcare delivery models.

**Abstract:**

Objective: To examine the association between frequency of mobile clinic visits and diabetes or hypertension control among patients who received regular mobile clinic care, controlling for patient sociodemographic characteristics and comorbidities. Design: Retrospective cohort study using patient chart review. Sample: Patients who regularly visited mobile medical clinics in Southern California (*N* = 218) between 1 January 2018 and 31 December 2019. Measurements: The dependent variables were hemoglobin A1c and blood pressure control. The independent variable was number of visits per year. Longitudinal associations were examined using a linear mixed model or generalized linear mixed model. Results: Among regular mobile clinic patients with diabetes (*n* = 86), there was no significant association between number of visits and hemoglobin A1c control (hemoglobin A1c < 6.5). Among regular mobile clinic patients with hypertension (*n* = 129), the odds of hypertension control (blood pressure < 140/90 mmHg) over time significantly increased as the frequency of clinic visits increased (adjusted OR = 5.27, 95% CI 1.63–16.99). Conclusions: The study findings suggest that regular mobile clinic use by adults with hypertension improves blood pressure control over time. However, the frequency of mobile clinic visits had no effect on diabetes control overtime. Patients with diabetes need additional interventions to achieve hemoglobin A1c control.

## 1. Introduction

More than 2000 mobile clinics operate in the United States, providing an estimated 5 to 6.5 million visits annually and serving more than 2.1 million uninsured people [1]. Historically, mobile medical clinics have served the most vulnerable populations in the nation, providing quality access to migrant workers, women in need of prenatal care, children with asthma, and minorities with HIV [2]. Mobile clinics are uniquely positioned to provide health care services to people with chronic illnesses [2,3] and they contribute to alleviating health disparities among vulnerable and underserved populations [4]. In 2017, 28 million people did not have health insurance. Of the 62 million who were unable to access primary care services, 43% were low-income and 28% lived in rural areas [5].

Access to health care, an important determinant of health, includes insurance coverage, health care services, and timely care. Lack of insurance is one of the most formidable barriers to care [6]. Social determinants of health (i.e., conditions in the environment where people live, work, play, and pray) affect a wide range of health and quality-of-life outcomes and risks [7]. These modifiable barriers to access can lead to unmet health needs, delays in receiving appropriate care, and preventable hospital admissions [8].

Research suggests that mobile clinics can improve health outcomes, increase patient quality of life, and reduce health care costs by reducing unnecessary emergency department visits or hospital readmissions [2,9]. For example, a study by Song et al. [10] found that patients who received screening and counseling from a mobile medical clinic had reductions in both systolic and diastolic blood pressures by 10.7 mmHg and 6.7 mmHg, respectively. These changes in blood pressure were associated with 32% and 45% reductions in risks of myocardial infarctions and strokes, respectively, which translated into a cost savings estimation of $235,254 over 30 months.

It is critical to better understand how innovative models of care can address these social determinants of health to improve health care access and reduce health disparities for people with health care needs. According to the 2019 Kaiser Foundation Hospital report, residents in Southern California report poorer values on several indicators of access to care compared to residents of the state overall [11]. For example, two San Bernardino County cities, Fontana and Ontario, have lower rates of primary care visits (66.6% and 66.8%, respectively) compared to the rest of the state (72.9%). Prevalence of diabetes is higher in the Fontana and Ontario service regions (11.5% and 10.2%, respectively), compared to the rest of California (7.3%). Type 2 diabetes is a chronic illness that impacts the lives of people worldwide and is projected to be the 7th leading cause of death in the world by 2030 [12]. The cost of diabetes is a substantial expense for individuals and governments; therefore, implementing population-based interventions that focus on prevention, early detection, and medication management to prevent disease progression is paramount to halting the continued surge of diabetes globally [12,13,14].

In addition, prevalence of hypertension is higher in San Bernardino compared to the rest of the State of California (32% versus 27%, respectively [15]. Hypertension is a major risk factor for cardiovascular illness. Lowering blood pressure has been shown to decrease the incidences of stroke, heart attack, and heart failure. Further, 1 in 5 Hispanic males have hypertension. However, hypertension is more prevalent in non-Hispanic Blacks (57%) than Hispanics (43.7% [16]).

For optimal treatment of chronic illness, patients need to make appropriate use of available health care, including regular follow-up. Various factors can affect health care use among patients. The Andersen Behavioral Model (ABM) is one of the most frequently used frameworks for explaining and predicting patient utilization of health care services and related outcomes [17,18,19]. According to the ABM, use of health services is a function of individual predisposition to use services, factors which enable or impede use, and the need for care [17,18,19]. The ABM feedback loop provides a holistic perspective of the determinants of health that can influence access to health care. This loop shows that health outcomes can affect subsequent predisposing, enabling, and need characteristics of the individual and their utilization of health services. Need characteristics, which are most proximal to service use, are medical conditions that prompt the importance of medical care.

Access to quality health care is essential to achieve improved health outcomes and to increase quality of life. Limited access to care impacts each individuals’ ability to reach their full potential and negatively affects their quality of life [20]. However, it is unknown to what degree the utilization of mobile medical clinics influences outcomes of treatment for chronic diseases, such as diabetes and hypertension, especially among vulnerable individuals in marginalized communities in California. Therefore, the aim of this study was to examine the association between frequency of clinic visits and diabetes and hypertension control over a 2-year period among regular users of a mobile medical clinic in Southern California, after adjusting for sociodemographic characteristics and comorbidities. We hypothesized that adults with well-controlled diabetes or hypertension will use mobile clinics more frequently than those without well-controlled conditions.

## 2. Materials and Methods

### 2.1. Study Design and Sample

This retrospective cohort study included patients who received regular care in four mobile medical clinic locations in Southern California from 1 January 2018 to 31 December 2019. The Well of Healing Mobile Medical Clinic is a no-cost, faith-based, primary care mobile clinic licensed by the State of California as a free mobile clinic in San Bernardino County. The clinic opened its doors in 2004 as a non-profit 501(c)(3) organization and provides health care access to uninsured and underinsured people who live in the Southern California communities of Fontana, Ontario, San Bernardino, and Muscoy, an unincorporated area. The no-cost health care services provided by the clinic include a physical examination and evaluation by either a physician or a nurse practitioner, point-of-care testing, medications, health education, and prayer [21]. Patient medical record charts were reviewed to determine eligibility based on predefined study inclusion and exclusion criteria. Chart review was conducted to confirm diagnosis of diabetes and/or hypertension, presence of clinic visits within the study period, and availability of relevant clinical data. Only patients meeting these criteria were included in the study.

**Figure 1:**
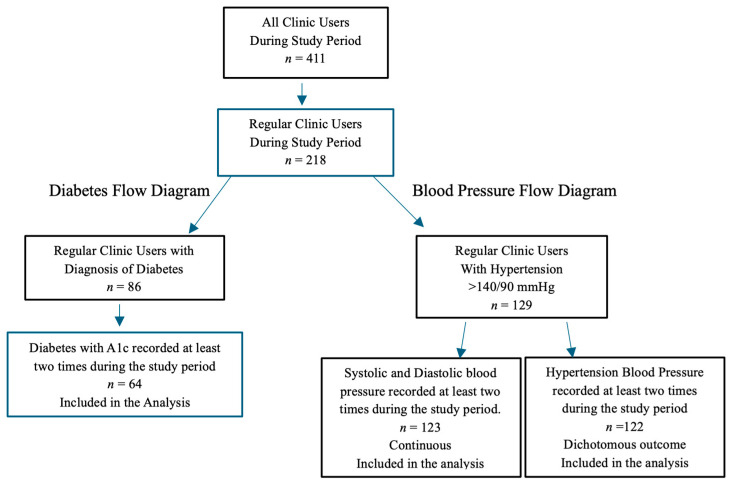
Diabetes and blood pressure data analysis flow diagram.

The study inclusion criteria included adults 26 years old and older who visited the clinic at least three or more times during the 2-year study period. Age 26 represents an important transition point in healthcare access in the United States, as many individuals lose eligibility for parental insurance coverage and may increasingly rely on services such as mobile clinics. For this reason, we included all adults aged 26 and older to ensure that the study reflected the full spectrum of patients receiving care rather than limiting the sample to older age groups. From a clinical perspective, individuals in this age range are more likely to have chronic illnesses of interest per our study criteria. A regular clinic user was defined as a person who visited the clinic three or more times per year, based upon the recommended visits for chronic illness reevaluation and management [22,23]. There were 411 patients who visited the clinic during the study period and 218 (53%) were identified as regular users. Of those regular users, 86 (39%) had a diagnosis of diabetes and 129 (59%) had a diagnosis of hypertension, with some patients having both conditions. Three patients (2%) had a documented handwritten diagnosis of hypertension, but blood pressure data were missing; therefore, they were excluded from condition-specific analyses. The number of new patients who were regular users during the study period was 58% (*n* = 127), whereas 42% were established patients (*n* = 91). New patients were defined as individuals documented in the chart as presenting to the mobile clinic for the first time during the study period. Figure 1 provides a diagram of the process through which patients were selected for the study and included in the analysis. Individuals who did not have at least two hemoglobin A1c measures were excluded from the diabetes control analysis. Individuals who did not have at least two blood pressure measures were excluded from the blood pressure and hypertension control analysis. Individuals with fewer than two A1c tests or blood pressure measurements during the study period were excluded because at least two values were required to support analysis of clinical patterns over time. Hemoglobin A1c and blood pressure were modeled as continuous variables, and all available measurements recorded during the study period were included as repeated measures rather than selecting a single value (e.g., mean, highest, or most recent) for each participant.

### 2.2. Data Collection

Approval of the study was granted by Well of Healing Mobile Medical Clinic Board of Directors. Data were retrieved by the lead author (A.C.) from the medical charts of all patients who visited the clinics during 2018–2019. A medical record data abstraction tool was created to capture information regarding the variables of interest from records of the patient’s initial visit and all subsequent visits between 2018 and 2019. All patients were assigned a subject ID number and deidentified data were entered into RedCap. Presence of diabetes was identified in the patient chart by record of disease diagnosis and/or prescription of diabetes medication (e.g., metformin, glipizide). For patients with diabetes, we collected all hemoglobin A1c readings during the 2-year study period. The presence of hypertension was identified in the patient chart by record of disease diagnosis and/or prescription of hypertensive medication (e.g., Amlodipine, HCTZ, Losartan, Lisinopril). For patients with hypertension, we collected all systolic and diastolic blood pressure readings during the 2-year study period.

### 2.3. Study Measures

The dependent variables in this study were hemoglobin A1c, systolic blood pressure, and diastolic blood pressure. Hemoglobin A1c and blood pressure were used as continuous variables and also dichotomous variables of controlled diabetes or hypertension were created. Control of diabetes was defined as a hemoglobin A1c level <6.5%, reflecting the clinical benchmark used at the mobile medical clinic. Although the CDC currently recommends an A1c goal of <7% for most individuals with diabetes [16], the <6.5% threshold was used to align with clinical practice at the study site. Hemoglobin A1c provides an average level of blood sugar over the prior 3 months; the higher the hemoglobin A1c level, the higher the average blood glucose levels over the past 3 months and, thus, the more poorly controlled the diabetes [24]. Hypertension was defined using the JNC-8 threshold of ≥140/90 mm Hg, consistent with the standard of practice at the study site [25]. Control of hypertension was defined as systolic pressure <140 mmHg and diastolic pressure <90 mmHg.

The main independent variable of interest was the number of clinic visits per year. For patients who were new to the clinic and had less than 1 year of study data, we calculated a projected number of visits per year, adjusting for the duration of observation in the study. We annualized visit frequency by dividing the total number of completed visits by the number of months observed and then multiplied by 12. This produced a projected annual visit rate that adjusted for differences in follow-up duration and allowed comparison across participants. Covariates were selected based on the ABM, including predisposing, enabling, and need factors. Predisposing factors comprised demographic characteristics, including sex (male/female), race/ethnicity (African American, White, Hispanic), age at first visit during the study period, and marital status (single, married). Enabling factors included insurance status (uninsured, private pay, public insurance) and housing status (housed, homeless). Need factors included obesity, depression, and the Charlson Comorbidity Index (CCI) score. The presence of depression and obesity was determined by chart review of diagnosis of depression or obesity or existing medication prescription for depression (Abilify, Elavil, Prozac, Zoloft based on known available treatment options). The CCI is a method of classifying comorbidity, which assesses the presence of 19 comorbidities (e.g., congestive heart failure, cerebral vascular accident, chronic obstructive pulmonary disease) and produces an unweighted count of the number of comorbid diseases and creates a sum that considers both the number (frequency) and seriousness (severity) of comorbid conditions [26]. The CCI is a validated and widely used tool for predicting mortality and cost of chronic illness [26]. Each chronic condition (diabetes and hypertension) was included as a covariate when analyzing outcomes related to the other condition to account for comorbidity. Baseline A1c and blood pressure values, when available, were included to adjust for initial disease severity. Follow-up A1c and SBP/DBP values were treated as the primary outcome measures.

### 2.4. Data Analysis

Descriptive statistics were used to summarize the baseline characteristics of the sample at the first visit during the study period and to describe the total number of visits by sample characteristics. Descriptive statistics were reported as mean and standard deviation for continuous variables, and as frequencies and percentages for categorical variables.

To analyze the continuous hemoglobin A1c data for diabetes patients and systolic and diastolic blood pressure data for hypertension patients during the study period we utilized a linear mixed model in lme4 v1.1.27.1 [27]. For dichotomous outcomes (controlled hypertension), we utilized a generalized linear mixed model with a logistic regression link. For hemoglobin A1c, modeling hemoglobin A1c as a continuous variable had the most powerful fit; therefore, we presented results using continuous hemoglobin A1c data. We first fitted a model with main effects. We then added an interaction term to the unadjusted model to examine the interaction between the frequency of clinic visits and the amount of time an individual had been in the study (referred to in the multivariate models as “study-time”) on hemoglobin A1c or blood pressure control. Several sensitivity tests were conducted to determine the best model fit. We used two-tailed tests with an alpha level of 0.05. The final multivariate model adjusted for all covariates of predisposing, enabling, and need factors based on the ABM theoretical model, regardless of statistical significance.

## 3. Results

### 3.1. Characteristics of the Study Participants

Table 1 presents the baseline characteristics of the 218 patients who received regular care and the number of visits between 1 January 2018 and 31 December 2019. These patients made a total of 1430 visits to the mobile clinics during the study period, with a mean number of 6.6 (*SD* = 4.2) visits. The majority of the regular mobile clinic users were female (68%), Hispanic (87%), and married (56%); mean age was 53 (*SD* = 11.0). Uninsured individuals accounted for 53% of regular users. Nearly all regular mobile clinic users reported a home address (99%), while only 1% indicated homeless housing status.

Table 2 presents the characteristics of patients with diabetes and hypertension. Of the regular clinic users, 39% had diabetes (*n* = 86), 59% had hypertension (*n* = 129), 26% had obesity (*n* = 57), and 2% had depression (*n* = 5). Among 86 mobile clinic patients with diabetes, 67% had uncontrolled diabetes (hemoglobin A1c > 6.5; *n* = 58). Of 129 regular users with hypertension, 54% had uncontrolled hypertension (*n* = 70). Nearly 73% of the study sample had other comorbidities (CCI ≥ 1).

### 3.2. Associations Between Number of Visits and Hemoglobin A1c Values

Table 3 presents the linear mixed model regression results examining the association between the number of mobile clinic visits per year and hemoglobin A1c levels over time among regular users with initial and follow-up hemoglobin A1c data (*n* = 64). These patients made a total of 618 visits to the mobile clinics, with a mean of 9.6 (*SD* = 3.0) number of visits.

In both unadjusted and adjusted models, no association were found between number of visits and hemoglobin A1c measurement (*p* > 0.05). Additionally, none of the predisposing or enabling variables were significantly associated with hemoglobin A1c level. Among need variables, CCI score was significantly associated with hemoglobin A1c levels over time in both the unadjusted and adjusted models. Compared to those with a CCI of 0, those with a CCI score of 2 had a 2.46 increase in hemoglobin A1c level (Coefficient = 2.46, 95% CI 0.15 to 4.77, *p* = 0.041), adjusting for patient sociodemographic characteristics and comorbidities. There was no interaction effect between study-time and number of visits on hemoglobin A1c control.

### 3.3. Associations Between Number of Visits and Blood Pressure and Hypertension Control

Table 4 presents linear mixed model results examining the association between number of mobile clinic visits and continuous measurement over time, while controlling for patient sociodemographic characteristics and comorbidities.

**Figure 2:**
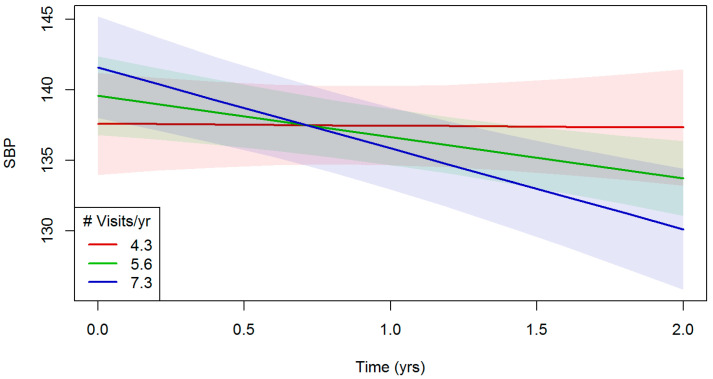
Systolic blood pressure (SBP) trajectory over the 2-year study period by number of visits among regular users of a mobile medical clinic in Southern California, from 1 January 2018 to 31 December 2019, after adjusting for sociodemographic characteristics and comorbidity. Note: Shaded areas indicate 95% confidence intervals. Note. Abbreviations: # = number, yr = year; yrs = years.

There was no evidence for a main effect of number of visits per year on systolic blood pressure, either in the unadjusted or adjusted models (*p* > 0.05). However, there was a significant interaction effect between number of clinic visits and study-time on systolic blood pressure in the unadjusted model (β = −9.48, 95% CI −16.8 to −2.12, *p* = 0.012). The significant interaction between number of clinic visits and study-time, on systolic blood pressure remained in the adjusted model (β = −10.76, 95% CI= −18.28 to −3.25, *p* = 0.005). Figure 2 shows the trajectory of systolic blood pressure over time by frequency of visits. The purple line, representing the most frequent clinic users (# visits/year = 7.3), shows the sharpest decline in systolic blood pressure over the 2-year study period, whereas the red line, representing the least frequent clinic users, shows almost no change in systolic blood pressure over time.

For diastolic blood pressure, both in the unadjusted and adjusted models, there was no evidence of a main effect of number of visits on diastolic blood pressure (*p* = 0.28) or an interaction effect with study-time. However, mobile clinic patients with obesity had an increase in diastolic blood pressure by 3.95 compared to those without obesity in the unadjusted model (β = 3.95, 95% CI 0.72 to 7.19, *p* = 0.018). Additionally, there was a significant inverse association between CCI and diastolic blood pressure control. Compared to those with a CCI score of 0, significant decreases in diastolic blood pressure control were observed for those with a CCI score of 2 (β = −6.89, 95% CI −11.40 to −2.42, *p* = 0.003), a CCI score of 3 (Coefficient = −8.62, 95% CI −13.30 to −3.93, *p* = 0.000), and a CCI score of 4+ (Coefficient = −14.5, 95% CI −21.20 to −7.82, *p* = 0.000).

**Figure 3:**
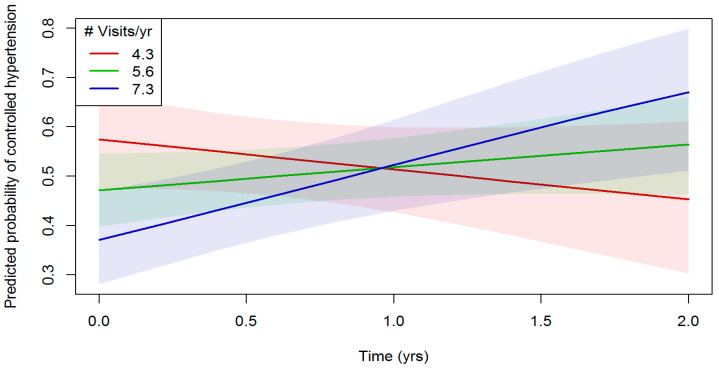
Predicting probability of hypertension control by number of visits over the 2-year study period among regular users of a mobile medical clinic in Southern California, from 1 January 2018 to 31 December 2019, after adjusting for sociodemographic and health characteristics. Note: Shaded areas indicate 95% confidence intervals. Note. Abbreviations: # = number, yr = year; yrs = years.

Table 4 presents results of a generalized linear mixed model, with a logistic regression link examining the relationship between the number of clinic visits and hypertension control (blood pressure < 140/90 mmHg). There was no evidence for a main effect of the number of clinic visits on hypertension control in the unadjusted and adjusted models (adjusted OR = 0.46, 95% CI 0.20 to 1.08, *p* = 0.075). However, there was a significant interaction between the number of clinic visits and study-time on hypertension control (OR = 4.91, 95% CI 1.54 to 15.60, *p* = 0.007) and the significant interaction remained in the adjusted model (adjusted OR = 5.27, 95% CI 1.63 to 16.99, *p* = 0.005). Figure 3 illustrates the probability of hypertension being controlled over time by frequency of visits. Patients with the highest frequencies of clinic visits (mean 7.3 visits/year in the purple line) showed increased hypertension control over the 2-year study period whereas patients with the lowest frequencies of clinic visits (mean 4.3 visits/year in the green line) showed decreased hypertension control over time. No predisposing, enabling, or need variables were significant in the unadjusted and adjusted models.

## 4. Discussion

In this retrospective cohort study, we examined two selected chronic illness biomarkers (hemoglobin A1c and blood pressure) over a 2-year period among regular mobile medical clinic users in Southern California. We hypothesized that adults with well-controlled diabetes or hypertension would have used mobile clinics more frequently than those without well-controlled diabetes or hypertension and that the number of clinic visits would influence the controlled status. This study’s findings suggest that the trajectory of hypertension control over time among regular mobile clinic users significantly differ by the frequency of clinic visits. However, such an interaction effect between the number of visits and the length of time a participant was engaged with the clinic was not observed for diabetes control.

In this study of regular mobile medical clinic users in Southern California, patients had high prevalence of hypertension (59%) and diabetes (39%) and the majority of patients were Hispanic, age around 60, uninsured, female patients with multiple comorbidities. These patient characteristics are similar to reports from other studies of mobile clinics [1,28,29] with the exception that other studies have demonstrated that Black patients visit mobile clinics more frequently than Hispanic patients [10].

This study did not find an association between frequency of clinic visits and diabetes control measured by hemoglobin A1c level among regular mobile medical clinic users. Although previous research suggests that increased access to medication improves healthcare outcomes for diabetes [30,31], more frequent visits to the mobile medical clinic for regular users in this study were not linked to improvement in hemoglobin A1c. In fact, many of the patients in this study showed poor diabetes control (hemoglobin A1c > 6.5), indicating challenges in adequate management of diabetes, particularly in this vulnerable population with limited resources. For adults with diabetes, only one in eight people achieve the American Diabetes Association goals for hemoglobin A1c [29]. The lack of association may reflect unmeasured factors such as adherence to medication, duration of disease and social determinants of health. These factors may influence glycemic control but were not captured in the available data. Therefore, futures studies need to identify modifiable factors that collectively can improve hemoglobin A1c values in individuals, particularly for vulnerable populations who depend on mobile clinics for health care services.

This study found that patients who visited the mobile clinic more frequently over time were more likely to achieve controlled hypertension. There has been little research that examined the relationship between healthcare utilization frequency and hypertension control, but previous literature provides evidence that mobile health clinics improve outcomes of treatment for hypertension over time. In a study by Song et.al. [10] mobile clinic patients with hypertension had an average reduction of 10.7 mmHg in systolic blood pressure and 6.2 mmHg in diastolic blood pressure compared to their first visit. These patients had a mean of six visits to the clinic during a thirty-month period [10]. Another study found that hypertension control was significantly associated with visit intervals to a primary care provider among middle and older populations [23]. The results of this study joins a growing body of evidence that regular and frequent care provided by mobile medical clinics can contribute to hypertension control among their patients.

## 5. Limitations

This study had several limitations to consider. First, the retrospective study design and use of existing medical record data limited control over available information and the consistency of the data collected. Education level and income may be important potential confounders, but information on those variables were not available in the patient medical charts. In addition, this study utilized medical records that were designed for clinical care, which were not systematically completed for research purposes and therefore contained gaps in data. In contrast, most prior studies reported only aggregate data and provided big picture insight regarding care [1]. Using medical record review, including biological data, can provide advantages over self-report.

Second, the clinic involved in this study was faith-based (non-denominational) and, given its religious affiliation, the population served may have chosen to visit the clinic because of its faith-based mission; alternatively, this could have been a deterrent to potential patients. Thus, the findings may not be generalizable to patients seeking care from other types of mobile clinics. However, future studies should consider the faith-based environment as an enabling factor for mobile medical clinic use.

Third, there was a lack of any measures of quality of care (e.g., nature of interactions with patients) and how this covariate could influence outcomes. Future studies should document the experience of adults receiving care aboard a mobile clinic as a way to measure quality of care and understand the patient perception of this medical delivery model.

Finally, this study included all patients who visited the clinics between 2018 and 2019, and the observation period varied by patient. In the study sample, 58% of patients were new to the clinic and some patients made only a few visits during the study period. Consequently, these variations were taken into consideration during the analysis, and we calculated a projected number of clinic visits per year using available data; using projected data in some cases may have introduced error. Additionally, the sample size for the diabetes cohort was modest, and a number of participants were excluded due to insufficient A1c data, which may have reduced statistical power and limited the ability to detect statistically significant associations.

## 6. Implications for Public Health Nursing

Public health nursing emphasizes the importance of understanding the dynamics of where people live, work, play, and pray. Mobile medical clinics are positioned to inform policy, improve chronic disease treatment outcomes, and advance health equity among vulnerable populations, and thus an ideal way to bridge the gap between hospitals and communities [1]. Future research is needed to investigate the cost of services aimed toward reducing the burden of chronic illness (e.g., diabetes type 2, hypertension) on patients, communities, and health systems.

Understanding the patient characteristics and health outcomes of individuals served by mobile medical clinics provides researchers, public health nurses, policymakers, and health systems leaders the data needed to guide clinical practice interventions in unconventional spaces, support non-traditional health care delivery models, and leverage policy change to increase health care access for vulnerable populations. Future studies should document the experience of individuals seeking regular care aboard mobile medical clinics, as a way to better understand their perspective of visit frequency in improving health outcomes in this population.

These research findings improve our understanding of the characteristics of people who regularly utilize care aboard mobile clinics and help us to ascertain the effects of non-traditional health care delivery models on health outcomes among medically underserved individuals. An exploration of financial models for sustaining these healthcare systems is essential to continuing the public health efforts focused on improving health care access and outcomes in individuals and communities.

## 7. Conclusions

Utilization of mobile medical clinics can contribute to reducing the burden of illness by providing consistent access to regular clinical care, particularly to vulnerable, marginalized populations. This study joins a growing body of evidence demonstrating that frequent utilization of mobile medical clinics can improve chronic illness control such as hypertension. For patients with diabetes, we did not find a significant association with frequency of mobile medical clinic visits and A1c levels, and this finding may indicate that diabetes control requires consideration of other aspects, particularly patients’ comorbidity conditions. Given this information, providers, health system leaders, and patients with diabetes should consider additional interventions for improving diabetes control.

Knowledge of the role of mobile medical clinics in improving chronic illness control and the importance of ensuring access as often as needed among individuals who visit mobile medical clinics gives health systems, policy makers, and government agencies a leg up in directing resources to the communities in their catchment areas when needing to reach vulnerable populations. Further, the results of this research add to the scientific foundation on which to develop future studies that focus on mobile clinic patients and inform care management interventions for individuals with chronic illness, such as diabetes and hypertension.

## Figures and Tables

**Table 1:** Baseline characteristics of patients receiving regular care at mobile medical clinics in Southern California between 1 January 2018 and 31 December 2019 by total number and mean number of visits.

Variables	Regular Mobile Clinic Users ^a^ *n* (%)	Total Number of Visits ^b^ *n*	Number of Visits ^c^ Mean (*SD*)
TOTAL SAMPLE *	218	1430	6.6 (4.2)
NEW PATIENTS *	127 (58%)	494	3.9 (2.9)
Predisposing variables			
Gender/Sex			
Female	150 (68.8)	985	6.6 (4.1)
Male	68 (31.2)	445	6.5 (4.4)
Race/Ethnicity			
Hispanic	190 (87.2)	1254	6.6 (4.2)
African American	10 (4.6)	43	4.3 (4.8)
White	3 (1.4)	32	10.7 (4.9)
Other/Unknown	15 (6.9)	101	6.7 (3.4)
Age			
25–35	11 (5.0)	27	2.5 (2.5)
36–45	43 (19.7)	240	5.6 (4.0)
46–55	68 (31.2)	436	6.4 (4.3)
56–65	70 (32.1)	510	7.3 (4.1)
66+	26 (11.9)	217	8.3 (3.7)
Mean (*SD*)	53.04 (11.10)	n/a	n/a
Marital status			
Married	122 (56.0)	818	6.7 (4.2)
Single	67 (30.7)	369	5.5 (4.3)
Unknown ^d^	29 (13.3)	243	8.4 (3.1)
Enabling variables			
Insurance status			
Uninsured	115 (52.8)	846	7.4 (4.2)
Unknown ^d^	93 (42.7)	530	5.7 (4.0)
Other ^e^	10 (4.6)	54	5.4 (3.9)
Housing status			
Housed	217 (99.5)	1429	6.6 (4.2)
Homeless	1 (0.5)	1	1.0 (NA)
Clinic location			
Ontario	102 (46.8)	622	6.1 (4.3)
Fontana	70 (32.1)	511	7.3 (3.6)
Muscoy	43 (19.7)	267	6.2 (4.6)
San Bernardino	3 (1.4)	30	10.0 (2.6)
Need variables			
Chronic illness			
Hypertension ^f^	129 (59.2)	1020	7.9 (3.9)
Diabetes ^g^	86 (39.4)	696	8.1 (3.9)
Obesity ^h^	57 (26.1)	464	8.1 (4.2)
Depression ^i^	5 (2.3)	53	10.6 (6.3)
Charlson Comorbidity index score ^j^			
0	60 (27.5)	264	4.4 (3.3)
1	59 (27.1)	377	6.4 (4.4)
2	54 (24.8)	410	7.6 (4.2)
3	35 (16.1)	296	8.5 (3.6)
4+	10 (4.6)	83	8.3 (3.8)

* Row percent; ^a^ Have 3+ visits per year; ^b^ Total number of visits = number of visits during the study period between 1 January 2018–31 December 2019; ^c^ Mean number of visits—mean number of visits over the 2-year study period with standard deviation. *SD* = Standard Deviation; ^d^ Unknown = Missing from record/not reported; ^e^ Other = Medicare only, Medicare and Medicaid, private pay, or Covered California; ^f^ Hypertension = Had a written diagnosis of hypertension in patient chart, systolic >140 and/or diastolic > 90 and/or prescription of hypertensive medication (e.g., Amlodipine, HCTZ, Losartan, Lisinopril); ^g^ Diabetes = Had a written diagnosis of diabetes in the chart, recorded hemoglobin A1c > 6.5, or was taking diabetes medication (e.g., metformin, glipizide); ^h^ Obesity = Had a written diagnosis of obesity; ^i^ Depression = Had a written diagnosis of depression or an existing medication prescription for depression (Abilify, Elavil, Prozac, or Zoloft based on known available mobile clinic treatment options) ^j^ https://www.mdcalc.com/charlson-comorbidity-index-cci (accessed on 18 November 2021).

**Table 2:** Baseline characteristics of patients receiving regular care at mobile medical clinics in Southern California between 1 January 2018 and 31 December 2019 by presence of uncontrolled diabetes or hypertension.

Variables	Diabetes Yes ^a^	Uncontrolled ^b^ Diabetes Hemoglobin A1c ≥ 6.5	Hypertension Yes ^d^	Uncontrolled ^e^ Hypertension Systolic ≥ 140 or Diastolic ≥ 90
TOTAL SAMPLE *	86 (39.4)	58 (67.4) ^c^	129 (59.2)	70 (54.3) ^f^
Predisposing variables				
Gender/Sex				
Female	55 (64.0)	37 (63.8)	88 (68.2)	48 (68.6)
Male	31 (36.0)	21 (36.2)	41 (31.8)	22 (31.4)
Race/Ethnicity				
Hispanic	77 (89.5)	51 (87.9)	114 (88.4)	62 (88.6)
African American	0 (0.0)	0 (0.0)	4 (3.1)	4 (5.7)
White	3 (3.5)	2 (3.4)	1 (0.8)	0 (0.0)
Other/Unknown	6 (7.0)	5 (8.6)	10 (7.8)	4 (5.7)
Age				
25–35	1 (1.2)	0 (0.0)	1 (0.8)	0 (0.0)
36–45	16 (18.6)	11 (19.0)	11 (8.5)	7 (10.0)
46–55	18 (20.9)	10 (17.2)	39 (30.2)	23 (32.9)
56–65	39 (45.3)	28 (48.3)	55 (42.6)	28 (40.0)
66+	12 (14.0)	9 (15.5)	23 (17.8)	12 (17.1)
Mean (*SD*)	55.5 (10.0)	56.4 (10.3)	57.4 (9.9)	56.5 (9.1)
Marital status				
Married	53 (61.6)	34 (58.6)	82 (63.6)	44 (62.9)
Single	20 (23.3)	13 (22.4)	29 (22.5)	17 (24.3)
Unknown ^g^	13 (15.1)	11 (19.0)	18 (14.0)	9 (12.9)
Enabling variables				
Insurance status				
Uninsured	52 (60.5)	37 (63.8)	78 (60.5)	45 (64.3)
Unknown ^g^	32 (37.2)	19 (32.8)	47 (36.4)	23 (32.9)
Other ^h^	2 (2.3)	2 (3.4)	4 (3.1)	2 (2.9)
Housing status				
Housed	86 (100.0)	58 (100.0)	129 (100.0)	70 (100.0)
Homeless	0 (0.0)	0 (0.0)	0 (0.0)	0 (0.0)
Clinic location				
Ontario	38 (44.2)	24 (41.4)	55 (42.6)	26 (37.1)
Fontana	32 (37.2)	24 (41.4)	45 (34.9)	23 (32.9)
Muscoy	14 (16.3)	8 (13.8)	26 (20.2)	19 (27.1)
San Bernardino	2 (2.3)	2 (3.4)	3 (2.3)	2 (2.9)
Need variables				
Chronic illness				
Hypertension ^a^	66 (76.7)	45 (77.6)	129 (100.0)	70 (100.0)
Diabetes ^d^	86 (100.0)	13 (22.4)	66 (51.2)	34 (48.6)
Obesity ^i^	24 (27.9)	58 (100.0)	39 (30.2)	19 (27.1)
Depression ^j^	1 (1.2)	0 (0.0)	3 (2.3)	2 (2.9)
Charlson Comorbidity Index score ^k^				
0	5 (5.8)	1 (1.7)	17 (13.2)	12 (17.1)
1	21 (24.4)	15 (25.9)	31 (24.0)	18 (25.7)
2	29 (33.7)	19 (32.8)	43 (33.3)	23 (32.9)
3	23 (26.7)	18 (31.0)	30 (23.3)	14 (20.0)
4+	8 (9.3)	5 (8.6)	8 (6.2)	3 (4.3)

* Row percent; ^a^ Diabetes Yes, Had a written diagnosis of diabetes in the chart, recorded hemoglobin A1c > 6.5, or was taking diabetes medication (e.g., metformin, glipizide); ^b^ Diabetes uncontrolled, hemoglobin A1c > 6.5 at baseline; ^c^ Hemoglobin A1c controlled, and uncontrolled numbers do not equal the number of patients with diabetes because hemoglobin A1c data was missing hemoglobin A1c recorded measure *(n* = 22); ^d^ Hypertension Yes, Had a written diagnosis of hypertension in the chart, systolic > 140 and/or diastolic > 90 and/or prescription of hypertensive medication (e.g., Amlodipine, HCTZ, Losartan, Lisinopril); ^e^ Hypertension uncontrolled; record of blood pressure > 140/90; ^f^ Hypertension controlled, and uncontrolled numbers do not equal the number of patients with hypertension because blood pressure data was missing at baseline *(n* = 3); ^g^ Unknown = Missing from record/not reported; ^h^ Other = Medicare only, Medicare and Medicaid, private pay and Covered California; ^i^ Obesity = Had a written diagnosis of obesity; ^j^ Depression = Had a written diagnosis of depression or an existing medication prescription for depression (Abilify, Elavil, Prozac, or Zoloft based on known available mobile clinic treatment options) ^k^ https://www.mdcalc.com/charlson-comorbidity-index-cci (accessed on 18 November 2021).

**Table 3:** Linear mixed model regression: Association between number of visits and diabetes among regular clinic users ^a^ at mobile medical clinics in Southern California between 1 January 2018 and 31 December 2019.

	Patients with Diabetes ^b^ & Baseline Hemoglobin A1c	Total Number of Visits ^c^	Mean Number of Visits ^d^	Hemoglobin A1c Value	
Variable	*n* (%)	*n*	Mean (*SD*)	Unadjusted Model Coefficient (95% CI)	Adjusted Model Coefficient (95% CI)
TOTAL SAMPLE	64 (100)	618	9.66 (3.0)		
Predisposing variables ^z^					
Gender/Sex					
Female	42 (65.6)	408	9.7 (2.6)	0.03 (−0.78, 0.84)	−0.02 (−0.93, 0.88)
Male	22 (34.4)	210	9.5 (3.8)	Referent	Referent
Race/Ethnicity					
Hispanic	56 (87.5)	545	9.7 (3.1)	0.87 (−0.26, 1.99)	0.62 (−0.59, 1.83)
White	3 (4.7)	32	10.7 (4.9)	Referent	Referent
Age [Mean (*SD*)]	56.5 (10.2)	618	9.7 (3.0)	0.74 (−0.03, 1.51)	−0.01 (−0.08, 0.06)
Marital status					
Single	15 (23.4)	147	9.8 (2.8)	0.49 (−0.44, 1.42)	0.63 (−0.40−1.66)
Married	36 (56.2)	355	9.9 (3.4)	Referent	Referent
Unknown ^e^	13 (20.3)	116	8.9 (2.2)	−0.09 (−1.24, 1.05)	0.12 (−1.09, 1.33)
Enabling variables					
Insurance status					
Uninsured	42 (65.6)	435	10.4 (2.5)	0.37 (−0.44, 1.18)	0.35 (−0.53, 1.23)
Other ^f^	2 (3.1)	19	9.5 (2.1)	Referent	Referent
Need variables					
Chronic illness					
Hypertension ^g^	49 (76.6)	471	9.6 (3.0)	0.31 (−0.58, 1.21)	−0.23 (−1.21, 0.75)
Obesity ^h^	18 (28.1)	182	10.1 (4.0)	0.18 (−0.66, 1.03)	0.17 (−0.72, 1.06)
Charlson Comorbidity Index score ^i^					
0	3 (4.7)	19	6.3 (1.2)	Referent	Referent
1	16 (25.0)	151	9.4 (3.9)	1.49 (−0.34, 3.33)	1.03 (−1.17, 3.23)
2	20 (31.2)	203	10.2 (3.2)	2.23 (0.43, 4.04) *	2.46 (0.15, 4.77) *
3	18 (28.1)	174	9.7 (2.4)	1.83 (0.01, 3.65)	2.05 (−0.40, 4.50)
4+	7 (10.9)	71	10.1 (2.0)	0.66 (−1.33, 2.65)	0.80 (−1.99, 3.59)
Clinic visit					
Log number of visits per year	n/a	n/a	n/a	0.28 (−1.26, 1.82)	−0.10 (−1.93, 1.72)
Log number of visits per year × study time (interaction)	n/a	n/a	n/a	−0.43 (−1.84, 0.98)	−0.34 (−1.76, 1.08)

Significance levels: * *p* < 0.01; Abbreviations: *SD*, standard deviation; IRR, incident rate ratio; CI, confidence interval; ^z^ Based on the Andersen Behavioral Model we used to guide this analysis; we included all predisposing, enabling, and need factors; ^a^ Regular users = Have 3+ visits per year; ^b^ Diabetes Yes, Had a written diagnosis of diabetes in the patient chart; recorded hemoglobin A1c > 6.5, or was taking diabetes medication (e.g., metformin, glipizide); ^c^ Total number of visits = number of visits during the study period between 1 January 2018–31 December 2019; ^d^ Mean number of visits—mean number of visits over the 2-year study period with standard deviation. *SD* = Standard Deviation; ^e^ Unknown = Missing from record/not reported; ^f^ Other = Medicare only, Medicare and Medicaid, private pay and Covered California; ^g^ Hypertension = Had a written diagnosis of hypertension in the patient chart; systolic > 140 and/or diastolic > 90; ^h^ Obesity =Had a written diagnosis of obesity; ^i^ CCI Score = https://www.mdcalc.com/charlson-comorbidity-index-cci (accessed on 18 November 2021).

**Table 4:** Linear mixed model regression and a generalized linear mixed model with a logistic regression link: Association between number of visits and blood pressure and hypertension control among regular clinic users ^a^ at mobile medical clinics in Southern California between 1 January 2018 and 31 December 2019.

	Patients with Hypertension ^b^	Total Number of Visits ^c^	Mean Number of Visits ^d^	Systolic Blood Pressure		Diastolic Blood Pressure		Hypertension Control (<140/90 mmHg)	
Variable	*n* (%)	*n*	Mean (*SD*)	Unadjusted Model Coefficient (95% CI)	Adjusted Model Coefficient (95% CI)	Unadjusted Model Coefficient (95% CI)	Adjusted Model Coefficient (95% CI)	Unadjusted Model OR (95%CI)	Adjusted Model OR (95% CI)
Total Sample	124								
Predisposing variables ^z^									
Gender/Sex									
Female	85 (68.5)	712	8.4 (3.6)	−2.85 (−7.54, 1.84)	−1.39 (−6.08, 3.30)	−2.69 (−5.99, 0.60)	−3.37 (−6.46, 0.28) *	1.18 (0.72, 1.95)	1.09 (0.65, 1.83)
Male	39 (31.5)	317	8.1 (4.0)	Referent	Referent	Referent	Referent	Referent	Referent
Race/Ethnicity									
Hispanic	109 (87.9)	908	8.3 (3.8)	−3.49 (−10.40, 3.39)	−1.865 (−8.74, 5.01)	−0.0165 (−4.87, 4.84)	−1.59 (-6.12, 2.95)	1.17 (0.56, 2.43)	1.18 (0.55, 2.50)
White	1 (0.8)	13	13.0 (NA)	Referent	Referent	Referent	Referent	Referent	Referent
Age									
Mean (*SD*)]	57.1 (9.6)	1029	8.3 (3.7)	0.302 (0.08, 0.52)	0.57 (0.20, 0.93) **	−0.4 (−0.541, −0.258) ***	−0.21 (−0.45, 0.03)	0.99 (0.97, 1.02)	0.96 (0.92, 1.00) *
Marital Status									
Single	26 (21.0)	217	8.3 (4.0)	0.46 (−5.09, 6.01)	−0.15 (−5.60, 5.29)	2.58 (−1.22, 6.37)	1.16 (−2.45, 4.76)	1.15 (0.641, 2.05)	1.06 (0.58, 1.95)
Married	80 (64.5)	645	8.1 (3.9)	Referent	Referent	Referent	Referent	Referent	Referent
Unknown ^e^	18 (14.5)	167	9.3 (2.4)	−2.4 (−9.85, 5.05)	−2.24 (−9.47, 5.00)	−6.51 (−11.6, −1.44) **	−3.09 (−7.90, 1.72)	1.33 (0.69, 2.56)	1.13 (0.57, 2.33)
Enabling variables									
Insurance Status									
Uninsured	74 (59.7)	664	9.0 (3.5)	1.71 (−2.80, 6.23)	1.44 (−2.98, 5.85)	0.818 (0.50, 1.35)	0.37 (−2.54, 3.27)	0.77 (0.48, 1.25)	0.85 (0.52, 1.39)
Other ^f^	4 (3.2)	28	7.0 (3.4)	Referent	Referent	Referent	Referent	Referent	Referent
Need variables									
Chronic illness									
Diabetes ^g^	66 (53.2)	549	8.3 (3.8)	−0.45 (−4.84, 3.95)	1.41 (−3.40, 6.21)	−0.762 (−3.86, 2.33)	1.00 (−2.19, 4.19)	0.98 (0.61, 1.55)	0.73 (0.43, 1.25)
Obesity ^h^	39 (31.5)	356	9.1 (4.0)	0.23 (−4.46, 4.91)	0.06 (−4.50, 4.62)	3.95 (0.72, 7.19) **	2.13 (−0.91, 5.16)	0.63 (0.39, 1.04)	0.69 (0.42, 1.14)
Charlson Comorbidity Index score ^i^									
0	17 (13.7)	130	7.6 (3.3)	Referent	Referent	Referent	Referent	Referent	Referent
1	30 (24.2)	242	8.1 (4.0)	5.03 (−2.37, 12.40)	2.06 (−5.69, 9.81)	−2.13 (−6.83, 2.56)	−0.77 (−5.87, 4.33)	0.95 (0.43, 2.10)	1.37 (0.58, 3.25)
2	40 (32.3)	348	8.7 (3.9)	5.07 (−2.00, 12.10)	−3.84 (−13.10, 5.41)	−6.89 (−11.40, −2.42) ***	−4.20 (−10.34, 1.95)	1.16 (0.55, 2.46)	2.45 (0.88, 6.87)
3	30 (24.2)	250	8.3 (3.7)	6.88 (−0.50, 14.30)	−4.11 (–14.84, 6.62)	−8.62 (−13.30, −3.93) ***	−4.84 (−11.92, 2.23)	1.00 (0.46, 2.20)	2.52 (0.77, 8.29)
4+	7 (5.6)	59	8.4 (3.0)	4.11 (−6.63, 14.80)	−10.45 (−25.55, 4.65)	−14.5 (−21.20, −7.82) ***	−8.05 (−18.09, 1.98)	1.42 (0.45, 4.54)	4.25 (0.77, 23.36)
Clinic visit									
Log number of visits per year	n/a	n/a	n/a	2.49 (−4.57, 9.55)	0.96 (−6.52, 8.44)	2.77 (−2.25, 7.79)	0.19 (−4.59, 4.97)	0.47 (0.21, 1.07)	0.46 (0.20, 1.08)
Log number of visits per year × study time (interaction)	n/a	n/a	n/a	−9.48 (−16.8, −2.12) **	−10.76 (−18.28, −3.25) **	−3.2 (−7.83, 1.44)	−4.21 (−8.87, 0.46)	4.91 (1.54, 15.60) **	5.27 (1.63, 17.00) **

Significance levels: * *p* < 0.01; ** *p* < 0.001; *** *p* < 0.0001; Abbreviations: *SD*, standard deviation; IRR, incident rate ratio; CI, confidence interval; ^z^ Based on the Andersen Behavioral Model we used to guide this analysis; we included all predisposing, enabling, and need factors; ^a^ Regular users = Have 3+ visits per year; ^b^ Hypertension = Had a written diagnosis of hypertension in the patient chart; systolic > 140 and/or diastolic > 90; ^c^ Total number of visits = number of visits during the study period between 1 January 2018–31 December 2019; ^d^ Mean number of visits—mean number of visits over the 2-year study period with standard deviation. *SD* = Standard Deviation; ^e^ Unknown = Missing from record/not reported; ^f^ Other = Medicare only, Medicare and Medicaid, private pay and Covered California; ^g^ Diabetes Yes, Had a written diagnosis of diabetes in the patient chart; recorded hemoglobin A1c > 6.5, or was taking diabetes medication (e.g., metformin, glipizide); ^h^ Obesity =Had a written diagnosis of obesity; ^i^ CCI Score = https://www.mdcalc.com/charlson-comorbidity-index-cci (accessed on 18 November 2021).

## Data Availability

The original contributions presented in this study are included in the article. Further inquiries can be directed to the corresponding author.
